# Problematic Online Dating: Systematic Review of Definitions, Correlates, and Study Designs

**DOI:** 10.2196/72850

**Published:** 2025-07-03

**Authors:** Marina F Thomas, Sylvia Dörfler, Gloria Mittmann, Verena Steiner-Hofbauer

**Affiliations:** 1 Research Centre Transitional Psychiatry, Karl Landsteiner University of Health Sciences, Dr. Karl-Dorrek-Straße 30, Krems, 3500, Austria, 43 2732 720904

**Keywords:** online dating, internet addiction, behavioral addiction, mobile apps, social media, compulsive behavior, smartphone addiction, problematic internet use, psychological distress, interpersonal relations

## Abstract

**Background:**

Users describe mobile dating apps as addictive, and researchers have attempted to formalize compulsive dating app use as a behavioral addiction. However, the concept of online dating addiction remains debated.

**Objective:**

This systematic literature review synthesized quantitative research on problematized online dating behaviors with a specific focus on (1) definitions and measurement of problematic dating app use, (2) the examined adverse correlates, and (3) study designs.

**Methods:**

From 16 databases, we identified 263 reports related to problematic online dating. Twenty-nine papers—published between 2009 and 2024—met inclusion criteria. They covered 32 quantitative studies investigating problematic dating app use. Sample sizes varied between 64 and 4057, and participant ages ranged between 13 and 80 years, while many participants were aged between 18 and 35 years.

**Results:**

Researchers problematized the following online dating behaviors (in descending order of frequency): use for certain motives (in 10 reports), problematic use in the sense of behavioral addiction (n=9), specific activities or experiences (n=9), compulsive use (n=6), a disbalance between offline and online interactions (too many or too few online interactions, n=4), and mere use or frequency (n=4). Even using dating apps for sexual motivations and relationship-seeking was linked to adverse correlates. Scholars have examined adverse correlates, including (1) mood and emotional issues (n=11), (2) anxieties (n=9), (3) user motives and other media variables (n=9), (4) undesired behaviors (n=8), (5) personality (n=8), (6) self-attitudes (n=7), (7) partner choice (n=5), (8) sexuality (n=5), and (9) interpersonal correlates (n=4). Methodologically, the most common scales (measuring use for certain motives and the 6-component behavioral addiction items) include life problems within their measurement of problematic dating app use (eg, use to forget problems and conflict due to use). Of 32 studies, only 3 were randomized experiments. All surveys measured dating app variables only at a single time point (cross-sectionally) and focused on between-person effects rather than within-person dynamics.

**Conclusions:**

Research on user motives dominates the field. To understand harmful media effects, researchers should measure media use and harmful consequences separately. However, motives are often worded as coping with an undesired state (eg, use to forget problems) or enhancing a desired state (eg, use for self-esteem enhancement). Similarly, behavioral addiction scales include life problems (eg, conflict due to use). These scales thus conflate predictor and outcome. Future literature reviews or meta-analyses that examine associations should include only results of scales that validly distinguish media use from its adverse outcomes. Overall, research on internet dating addiction—and internet addiction in general—requires theoretically grounded definitions as well as experimental and longitudinal studies modeling between- and within-person effects.

## Introduction

### Correlates of "Problematic" Media Use

There is a popular, academic, and clinical debate on the question of whether using new technologies at all, too much, or in maladaptive ways causes life problems. Different roots of the problem have been declared: some researchers are concerned about an entire technology (internet addiction, technology addiction, or digital addiction) [1], others about devices (compare computer addiction or problematic smartphone use) [2], specific apps (most prominently social media) [3], their engaging platform features (eg, likes and loot boxes) [4], or certain platform-enabled activities (binge watching, instant messaging, notification checking, pornography or news consumption, online shopping, gambling, gaming, or binge swiping) [5].

Opinions also differ on the question of which dose makes the poison. The most basic research problematizes any use of a certain technology or activity and compares users’ and nonusers’ well-being [6,7]. Others assume increasing psychological problems with increasing use or after some cutoff in usage frequency [8]. Behavioral addiction researchers, however, emphasize that heavy use does not equal problem use [9]. Others, in turn, problematize certain ways of using media (eg, browsing on social media) [10]. Many scholars criticize unregulated use: Research on addictive pornography use is immensely popular [11], and the number of studies on social media addiction grows exponentially [12]. In the last years, harms related to the use (or problem use) of mobile dating apps have also gained scientific momentum [13].

Dating apps have an ambivalent reputation. On the one hand, they are supposed to quickly connect strangers; on the other hand, users spend most of their time with in-app activities instead of initiating interactions [14]. Users report browsing through (ie, swiping) hundreds of profiles for up to 4 hours a day [15]. Frequent users are more likely to report problems regulating their swiping [16]. Psychoanalytic scholars even argue that the main function of the apps is to engage users’ desire for human connection and turn it toward the app [17]. Hence, dating apps are said to have turned dating into an addiction [18,19]. Comparable to other types of media, most users of dating apps do not report problem use [20]. Still, problem use is a topic with theoretical and societal relevance.

Scholars have attempted to formalize problematic dating app use in the sense of behavioral addiction [21]. Empirically, a body of literature linking problem use of dating apps to well-being starts emerging [21]. However, the field of problematic media use suffers from a range of theoretical ambiguities and methodological problems. Therefore, using the case of dating apps, the objective of this review is to systematize (1) definitions and assessments of problematized uses, (2) the examined undesired correlates, and (3) the employed methodological study designs.

### Problematized Online Dating

It remains debated how media problem use is defined. This review employs the overarching terms “problem use” and “problematized uses.” Some researchers problematize specific platform-facilitated activities (ie, profile browsing [22]). Few scholars test whether having used a certain media platform (or affordance) is a predictor of ill-being [23]. Yet, in the rather young field of dating app research, there are still studies comparing the well-being of lifetime users to that of nonusers [6]. Others problematize “usage frequency” (high involvement) [8]. Time spent online is usually retrospectively estimated and self-reported, but researchers have recently started using apps that monitor actual time spent [24]. Habitual use denotes use out of unconscious, conditioned habit, which can be inhibited if necessary [25]. Lifetime, frequent, and habitual use should not be considered problem uses to avoid overpathologizing common behaviors [26].

Other problematized uses are (1) excessive, (2) compulsive, and (3) problematic. First, excessive use means losing track of time or investing more time than originally intended [27,28]. Second, compulsive actions are defined as individuals’ continuation of “doing the same action because they feel like they have to, even though they know these actions do not align with their goals” [29]. However, the definitions are not clear-cut, and measures of excessive use can also contain items on losing control [16,28].

Third, problematic media use has been defined in various ways, with one meaning being addiction (seldom: dependency) following the style of problematic substance use. On the one hand, dependency or reliance on a medium is not considered pathological in media studies, but a normal consequence of satisfied communication needs [30]. On the other hand, in media and clinical psychology, there is “conceptual confusion surrounding this emotion-laden term” [30]. The dominant definition is the 6-component model of behavioral addiction [31]: The author argued that what is similar between addictive behaviors and substance use disorders are the 6 factors—salience, mood modification, tolerance, withdrawal, conflict, and relapse. Using 7 criteria (the 6 components [31] and problems), the authors [9] distinguished between addicted users, problem users, and highly engaged users. They categorized gamers who endorse all 4 of the core addiction criteria (relapse, withdrawal, conflict, and problems) as “addicted,” those who endorsed 2 or 3 of the core criteria as “problem users,” and those who endorsed all 3 peripheral criteria (salience, tolerance, and mood modification), but not more than one of the addiction criteria, as “highly engaged users.”

However, other definitions of problematic media use are employed. Caplan [27] proposed 7 subscales of problematic internet use, namely mood alteration, perceived social benefits (online compared to face-to-face communication), perceived social control (perceiving increased social control online), withdrawal (perceived difficulty of nonuse), compulsivity (difficulty controlling, guilt about use), excessive use (self-perceived overuse, losing track of time), and negative outcomes (life problems due to use). Another scale included the factors intrusion (longer use than intended, neglecting tasks), escapism (mood repair), and attachment (upset if use is impossible), and has been applied to problematic internet, television, and mobile phone use [30]. Due to these heterogeneous kinds of media problem use, this review aims to answer the following research question for the case of dating apps: how is problem use defined and *measured?*

### Adverse Correlates of Problematized Online Dating

The range of variables that have been linked to media (problem) use is long. As predictors of problem use, researchers have conceptualized user motives [32] or family addiction history [33]. As outcomes, researchers examined, for example, cybervictimization, social support [10,34], self-control, anxiety, depression, self-esteem [1], sleep quality [35], and undesired academic, social, or sexual behaviors [7,25]. Apart from the typical harmful media effects, research problematizing online dating may examine risks related to dating such as deception, sexualized harassment or abuse (physical and digital), unwanted pregnancy, or sexually transmitted diseases. In addition to media risks and dating risks, dating app researchers may investigate harms (eg, objectification and commodification) related to market-like affordances (eg, choice abundance and filtering).

Findings are mixed and depend on problematized uses, research methods, and study population [8]. The few existing longitudinal investigations have linked problematic dating app use to decision fatigue [36] or emotional exhaustion [37] over time. Since nonsignificant findings are less likely to be published [38], we inventoried which variables have been examined in relation to the problem use of dating apps, independent of whether associations were significant or not, and aimed to answer the following research question: which variables are studied as adverse outcomes?

### Study Designs

Cross-sectional and longitudinal surveys cannot rule out that, instead of media causing distress, already distressed individuals are drawn to media. Research on harmful consequences of media (problem) use should employ experimental designs to assure that media use and not preexisting differences between individuals cause group differences in distress. However, reviews on social media use and addiction [12] as well as on problematic smartphone use [39] found that most designs were cross-sectional. Likewise, a meta-analysis on internet use and depression indicated that almost all studies were cross-sectional [8]. Experiments manipulating (problem) media use are scarce [32,40].

Furthermore, individuals show differential susceptibility to media (problem) use, and between-person differences may not be as consequential as differences in media use compared to one’s own usual use [10]. Therefore, experimental and longitudinal studies should model both between- and within-person effects. When it comes to media use, many studies rely solely on between-person comparisons [10]. Therefore, we pose the following research question for the case of problem use of dating apps: which methodological study designs are employed to test associations between problem use and adverse outcomes?

## Methods

### Literature Search

The research questions and methods, including eligibility criteria, were registered on PROSPERO (International Prospective Register of Systematic Reviews; CRD42024601803) [41]. The search term read: ((AB (“problematic use” OR patholog* OR maladaptive OR excessive OR compuls* OR impulsive OR obsessi* OR addict*)) AND (AB (“dating app” OR “dating apps” OR “mobile dating” OR “online dating” OR “internet dating” OR “geosocial networking app” OR matchmaking OR swiping OR tinder OR grindr OR “match.com” OR okcupid OR jack’d OR badoo OR “partner choice” OR “partner search”))). Keywords had to be present in the abstract. We conducted the search on October 15, 2024. In a first step, we used EBSCOhost to simultaneously search Academic Search Index, MEDLINE Ultimate, Complimentary Index, PsychInfo, Directory of Open Access Journals, Business Source Index, Springer Nature Journals, Supplemental Index, Psychology and Behavioral Sciences Collection, Journals@OVID, JSTOR Journals, and ERIC. We filtered results to only include peer-reviewed papers; no other filters were applied. Afterward, we searched PubMed, Web of Science, ScienceDirect, and ACM Digital Library. As a second step, we searched the reference lists of included studies. For inclusion and exclusion criteria, see Table 1. For more information, see Multimedia Appendix 1 [15,16,20,42-74].

**Table 1.** Inclusion and exclusion criteria for our systematic review of problematic online dating definitions, correlates, and study designs.

Category	Inclusion criteria	Exclusion criteria
Topic	Maladaptive uses of mobile dating apps or online dating services	Studies comparing users to nonusers
Samples	All genders, all sexual orientations, all relationship statuses, and nonusers (eg, in experiments)	No restrictions
Article type	Quantitative empirical research	Nonempirical or qualitative research, case studies
Language	English and German	All other languages
Context	Any	No restrictions

### Literature Selection

Two authors (MFT and SD) individually screened titles and abstracts (full text where necessary) and included articles that researched maladaptive uses of mobile dating apps or online dating agencies (eg, excessive use) or specific activities typically performed on these platforms (eg, dating profile evaluations and online partner seeking) as well as adverse correlates of problematic use (eg, personality, user motive, mental health, or sexual health correlates). Cases of disagreement were discussed until an agreement was reached. All reports were published in English, peer-reviewed, and reported on empirical findings.

### Data Extraction and Synthesis

To ensure a systematic approach to data extraction, we employed a predefined data extraction form, which the authors had previously agreed upon. This form was designed to capture key study characteristics, including article characteristics (authors, title, country of origin, and year of publication), sample characteristics (age, gender, sexual orientation, user status, and sample size), method characteristics (methodological study design and definition of problematized uses of dating apps and used measures), and results characteristics (adverse correlates of problematized use). The form was applied consistently across all included studies to ensure reliability in the data extracted. The 2 researchers, MFT and SD, extracted the data and regularly discussed their findings to ensure that a consistent approach was maintained.

Once the relevant data were extracted, the features of definitions and adverse correlates of problematic dating app use, as well as the study designs, were synthesized using a descriptive analysis approach. Recurring themes and patterns were identified and quantitatively summarized. The themes, including the summarized features, were initially developed by one researcher (MFT) and subsequently discussed within the research team until we reached a consensus for all categories.

## Results

### Literature on Problematic Online Dating

**Figure 1.**
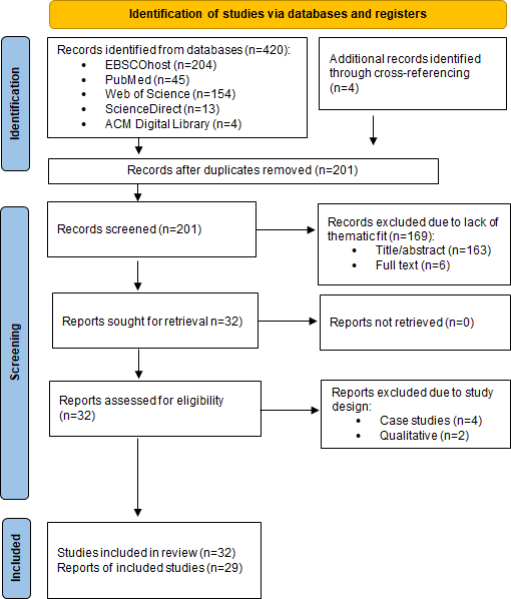
PRISMA (Preferred Reporting Items for Systematic Reviews and Meta-Analyses)–based flowchart of literature selection for our systematic review of problematic online dating definitions, correlates, and study designs.

The process and results of literature selection are visualized in Figure 1. Consequently, we summarize the features of included studies and synthesize problematized uses and adverse correlates of problem uses.

### Features of Included Reports

We included 29 published reports reporting on 32 studies (ie, samples). Three papers [15,51,61] each reported on 2 data collections. Two papers relied on the same data [20,63], and a third paper [64] also relied on this data collection but additionally included nonheterosexuals.

The included reports were published in English between 2009 and 2024. Table S1 in Multimedia Appendix 1 outlines each report’s country and study population including sample size, age, gender, sexual orientation, user status, and relationship status. Most samples stemmed from the United States. Sample sizes ranged from 64 to 4057, with participants aged between 13 and 80 years, while many samples were aged between 18 and 35 years mirroring the populations of both online daters and university students. Of 32 samples, 19 had relatively balanced gender ratios (40%‐59% male). Nine samples were female-dominated (0%‐39% male), and 4 were male-dominated (60%‐100% male).

### Problematized Online Dating

#### Overview

**Figure 2.**
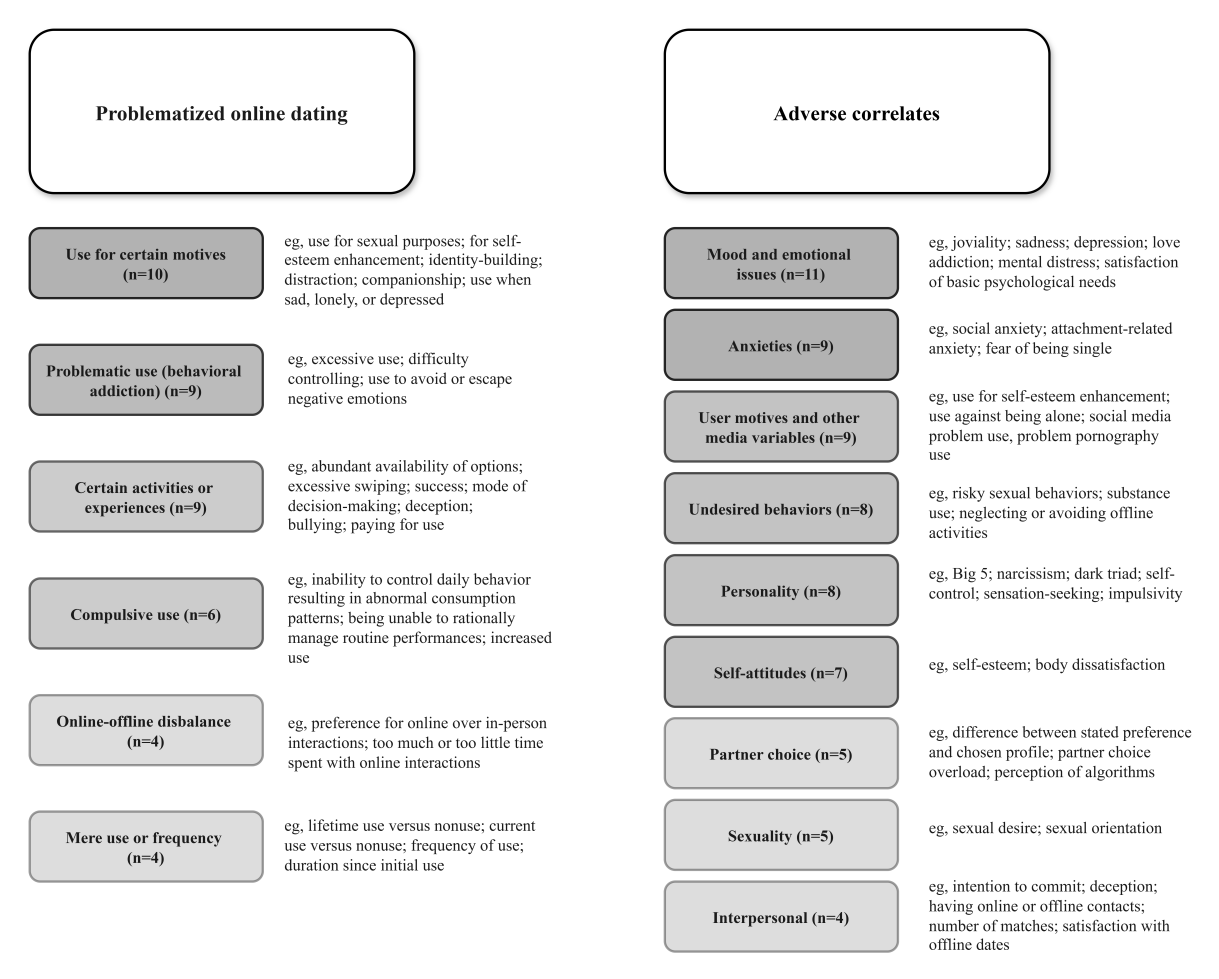
Overview of problematized online dating behaviors and adverse correlates across 29 papers (published 2009‐2024).

The first research question was how problem use is defined and measured. Each report’s definition of problem use and corresponding measurement is listed in Table S2 in Multimedia Appendix 1. Figure 2 shows an overview of problematized uses. We hereunder synthesize results quantitatively. We list all examined problem uses independent of whether associations were found significant. Results are presented in order, starting with the most used definition and proceeding to the least used definition of problematic dating app use.

#### Use for Certain Motives

A total of 10 reports defined problem use as use for certain (maladaptive) motives [42,44,48,49,53,56,57,59,62,64]. Two papers distinguished between use for either sexual or romantic motivations and grouped users into 2 categories (sexual versus all other purposes) based on a single dichotomous item asking, “If you use any of these [online dating] platforms, have you used them to have sexual encounters with others?” [57,59]. Other papers [48,59] measured motives with items by Sumter et al [68], with the Tinder Motives Scale [71], or using custom items [44]. Researchers problematized app use for self-esteem enhancement [56], identity building, and as a distraction [44]. It is noteworthy that harm was also associated with use for relationship seeking [53] or for companionship purposes [44,56], making these motives seem maladaptive for dating app use. Similarly, app use when lonely, sad, or depressed; when drinking alcohol; when using drugs; and to arrange sexual encounters in exchange for money [49] were tested as predictors of ill-being. Lastly, authors [62] problematized using dating apps with certain body ideal preferences (eg, for thinness and muscle tone).

#### Problematic Use in the Sense of Behavioral Addiction

Nine papers [20,46,47,50,52,56,60,63,64] conceptualized problem use of dating apps as behavioral addiction. Scholars consider problematic use of dating app “a behavioral addiction – whether a distinct one or a variant of sex-based addiction or Internet addiction. It could, indeed, also conceivably meet criteria for an impulse control disorder” [20]. Others cite the 6-component model and state that problematic use of dating apps and other technologies is characterized by “excessive use (e.g. more than a person intended to use), difficulty controlling use, use to avoid or escape negative emotions, and use that creates distress and functional impairment in various life domains” [56].

Six papers [20,50,52,60,63,64] employed the Problematic Tinder Use Scale (PTUS) [21] or later the Problematic Online Dating Apps Use Scale (PODAUS) [50] because these scales have 6 items covering exactly the 6 components put forward in the 6-component model of addiction [31]. Example items of the PTUS are “During the last year, how often have you spent much more time on Tinder than initially intended?” or “During the last year, how often have you tried to cut down on Tinder use without success?” [64].

Two more papers used similar scales [47,56]. The Bergen Facebook Addiction Scale [75] was used by Jayawardena et al [56], replacing “Facebook” with “Grindr.” The scale contains 6 items based on the 6-component model of addiction (2005). For example, the item on tolerance reads, “Felt an urge to use Facebook more and more,” and the conflict item reads, “Used Facebook so much that it has had a negative impact on your job/studies?” [75]. The Internet Addiction Test [70] was used by Drouin et al [47]. It contains 20 items on the 6 factors: excessive use, salience, lack of control, anticipation, neglecting work, and neglecting social life. Example items are “Do you choose to spend more time online over going out with others?” or “Do you try to cut down the amount of time you spend online and fail?” [70].

The ninth paper by Ding et al [46] employed the term problematic use and defined it as experiencing a “negative impact on mental health, social adjustment, and daily life as a result of excessive use.” Specifically, they employed a Chinese scale by Jiang [69]. It contained 5 dimensions, namely increased viscosity, physiological damage, misplaced anxiety, cognitive failure, and guilt*.*

#### Specific Activities or Experiences

Also, 9 papers were concerned with specific activities or experiences on dating apps, although their focus was diverse [15,16,20,43,47,53,60,65,67]. Since the central activity in online dating is swiping through profiles, 3 articles have specifically problematized the abundant availability of partnering options and manipulated them in experiments [15,43,65]. A fourth article has problematized excessive swiping [16] and measured it by adapting items on excessive smartphone use [28], for example, “I just have to keep swiping – there’s no other way.” The same study also problematized the mode of decision-making while swiping [16].

One paper examined deception in online dating [47], another online bullying victimization and perpetration [67], and another [58] examined trolling. Trolling included deceptive, aggressive, and disruptive behaviors, measured by the adapted Global Assessment of Internet Trolling [73]. The adaptation of March et al [58] reads, for example, “I enjoy griefing other people who access the app” or “The more beautiful and pure a thing is, the more satisfying it is to corrupt.”

Furthermore, Mignault et al [60] problematized chatting with many at the same time and meeting many new partners (and meeting them after chatting briefly). In contrast, others [48,66] defined having many matches and chat conversations as subjective online success and related it to desired emotional reactions. Having few contacts was an item of self-conscious social comparisons with other Tinder users (eg, “I think that most Tinder users have more matches than me”) by Her and Timmermans [53]. Similarly, Rochat et al [20] problematized a variable that combined the number of online and offline contacts, Tinder satisfaction, and motives (internal consistency not reported). In the same paper, they also looked at the correlates of paying for Tinder (comparing paying to nonpaying users). The authors constructed their own variables and used custom scales with unknown validity.

#### Compulsive Use

The compulsive framework was used in 6 papers [45,48,53-55,59]. Compulsive use of dating apps is defined as the “inability to reasonably control their daily behavior, resulting in abnormal consumption patterns” [48]. Following Dhir et al [72], Her and Timmermans [53, p. 1305] defined compulsive as being “unable to rationally manage his/her routined performances.” Yet, researchers examining compulsive use also problematized increased time spent online, while reducing time spent engaging in face-to-face interactions [45]. Thereby, individuals may “become dependent on the applications for an increasing number of interpersonal interactions” [45, p. 6].

Of the 6 papers, half [48,53,55] used the compulsive social media use scale [72]. This scale has 4 items (which originally referred to Facebook [FB]): “Spent a lot of time thinking about FB or planned use of FB?,” “Felt an urge to use FB more and more?,” “Used FB in order to forget about personal problems?,” and “Become restless or troubled if you have been prohibited from using FB?” Apart from this scale [72], 2 older scales for compulsive use were employed: Coduto et al [45] used 3 items adapted from Caplan [27]: “I want to, or have made unsuccessful efforts to, cut down or control my use of the Internet,” “I have attempted to spend less time online but have not been able to,” and “I feel guilty about the amount of time I spend online.” Thus, in addition to lack of control (or relapse), the scale by Caplan [27] measures guilt about spending high amounts of time.

Marciano et al [59] used another scale for compulsive use, namely the Compulsive Internet Use Scale (CIUS) [74]. This scale was built after analyzing the *DSM-IV* (*Diagnostic and Statistical Manual of Mental Disorders, Fourth Edition*) criteria for dependence and obsessive-compulsive disorder and the literature on behavioral addictions [74]. Marciano et al [59] used 14 items such as “Do you find it difficult to stop using dating apps?,” “Do you think you should use dating apps less often?,” and “Do you feel restless, frustrated, or irritated when you cannot use dating apps?”

#### Disbalance Between Offline and Online Interactions

Four articles problematized a disbalance between offline and online interactions [45,51,55,60]. Half of them were concerned about a preference for online over in-person social interactions [45,55] measured with 4 items. Two items indicated a preference, for example, feeling safer starting a conversation and more confident on dating apps than in face-to-face dating situations [45,55]. The 2 other items indicated that one is treated better on dating apps than offline, for example, “I’m treated better on dating apps than offline” [45].

A third study considered it problematic when users invested too little time in online interactions: The authors of one study [51] hypothesized that quickly meeting in person would be associated with increased sexual risk behavior. Similarly, another study [60] included one item about quickly meeting people offline. Thus, from their perspective, it seems preferable to invest a considerable amount of time (eg, a month or more) in online interactions.

#### Mere Use or Frequency

Four articles problematized mere use or frequent use [42,51,62,66]. Three papers relied on a single dichotomous item asking for use versus nonuse. Specifically, they distinguished nonusers from those who reported lifetime experience with dating apps [42,62] or from those who reported current use [51]. One study [66] measured frequency (from 1=never to 7=always) and analyzed the highest value (out of several social media platforms) of media use frequency (daytime and presleep). In their variable Tinder use pattern, the authors [20] combined different motives and experiences (we categorized this variable under Specific Activities or Experiences; see above) and also had an item asking since when individuals were using Tinder (duration since initial usage).

### Adverse Correlates of Problematized Online Dating

#### Overview

Table S3 in Multimedia Appendix 1 lists what each report tested as adverse correlates of problem use (independent of whether associations were significant or not). Figure 2 provides an overview. Note that researchers usually examine more than one outcome per study, so the numbers do not add up to the number of papers (n=29).

#### Mood and Emotional Issues

Eleven papers were concerned with joviality, happiness, sadness [48,53,55], negative mood [62], mental distress [59], depressive mood [20,56,57,63], suicidal ideation, and internalizing symptoms [57]. Other emotional issues were alexithymia [46], love addiction [50], and satisfaction and frustration of basic psychological needs [61].

#### Anxieties

A form of anxiety, for example, social or attachment-related anxiety, or fear of being single, was examined in 9 papers. These were 4 papers [15,16,45,46] as well as 5 papers that additionally examined mood and emotions [48,53,56,57,63].

#### Motives and Other Media Variables

Nine papers examined media use behaviors as correlates of dating app problem use: Five of these examined *user motives* of dating apps [20,48,56,61,63], for example, use for self-esteem enhancement [56,61], use to escape negative emotions, or use against being alone [56]. Two other papers associated dating app problem use with maladaptively using other media, namely problematic cyberpornography use, problematic social media use [50], and social media addiction—defined as excessive and irrational use of social media [66]. One paper examined the preference for online social interactions as a predictor of compulsive use [55], and another one looked at satisfaction with app use [64].

#### Undesired Behaviors

Of 8 papers examining undesired behaviors, 4 examined risky sexual behaviors, for example, the number of lifetime hookups, unprotected sex with multiple partners, or sex with someone just met [49,51,57,60]. Two papers were concerned with neglecting or avoiding offline activities such as class or work [45,46] in association with dating app problem use. Two papers examined decreased sleep quality [57,66], one study assessed substance use [57], and another assessed urges for disordered eating [42].

#### Personality

Eight papers related problem use to personality correlates: 3 papers selected the Big 5 [44,50,61], while 4 papers examined self-control, sensation-seeking [63], and (dysfunctional) impulsivity [20,51,58]. Two papers examined narcissism [58,67], Machiavellianism, psychopathy, and sadism [58].

#### Self-Attitudes

Self-attitudes were the topic of 7 papers, specifically self-esteem [15,61,63], body dissatisfaction, appearance-based rejection sensitivity or body esteem [52,62], upward social comparison on dating apps [16], and internalized homophobia [59].

#### Partner Choice

Five papers examined undesirable decision-making outcomes such as number of options searched, selectivity, a difference between stated preference and chosen profile [43,65], partner choice overload [15,16], or the perceptions that algorithmic recommendation systems are useful or that they restrict user choice [54].

#### Sexuality

Five papers examined variables related to sexuality, namely sexual desire [52,63], compulsive sexual behavior [57], cognitive distraction during sex, sexual esteem, sexual preoccupation, and sexual depression [52]. Another paper related sexual orientation and biological sex [44] to dating app problem use.

#### Interpersonal Correlates

Four articles considered interpersonal outcomes such as the intention to commit to a romantic partner [54], deception, perception of others’ deception [47], having online or offline contacts, number of current matches [63], and satisfaction with offline dates [64].

### Study Designs

Table S4 in Multimedia Appendix 1 lists each report’s study design. We included 29 reports. Since 3 reports [15,51,61] were 2-study papers, the 29 papers reported on 32 studies in total. Of the 32 studies included in the review, the majority (n=29) were surveys. All of the surveys were cross-sectional. Although one survey [62] used an ecological momentary assessment, they only assessed dating app variables (lifetime use and dating preferences) at one time point. Three studies were randomized experiments, of which 2 were field experiments using real profiles [43,65] and 1 used artificial bogus profiles [15].

## Discussion

### Principal Results

There is debate on the question of whether using new media frequently or maladaptively is associated with adverse psychological correlates. This review aimed at providing an overview of problem uses of dating apps, their undesired correlates, and the employed methodological study designs. We reviewed 29 scientific papers reporting on 32 studies. First, scholars problematized very different uses of dating apps ranging from (1) use for certain motives, (2) problematic use (behavioral addiction), (3) specific activities or experiences, (4) compulsive use, (5) online-offline disbalance, and (6) mere use or frequency. As adverse correlates, scholars examined, from most to least frequently: (1) mood and emotional issues (n=11), (2) anxieties (n=9), (3) user motives and other media variables (n=9), (4) undesired behaviors (n=8), (5) personality (n=8), (6) self-attitudes (n=7), (7) partner choice (n=5), (8) sexuality (n=5), and (9) interpersonal correlates (n=4*).* Regarding study designs, most studies (n=29) were correlational surveys and only 3 studies were experiments. All studies measured dating app variables only at a single time point and tested between-person effects.

### Comparison With Prior Work

#### Problematized Online Dating

First, motives are the most examined topic in dating app research. This is in line with communication scientists’ interest in individual uses and gratifications of media use [44]. Users report a number of motives ranging from relationship seeking to passing time [68]. Interestingly, dating apps are nominally intended to facilitate sexual and romantic interactions, and yet, using them for sexual motivations [57,59] and relationship seeking [53] were both linked to adverse correlates. So, scholars investigated if (and sometimes found that) using dating apps for what they are seemingly intended for is problematic.

Some of the examined motives described compensational uses such as self-esteem enhancement [56]. Use out of ill-being suggests that individuals turn to certain media and use them in maladaptive ways to cope with existing psychological issues. Logically, using technology to cope with ill-being cross-sectionally correlates with ill-being, so longitudinal and experimental designs should test whether this compensation is effective. Moreover, research should focus on psychological problems and functional impairments, instead of pathologizing everyday (media) behaviors [20].

Second, scholars often examined problematic use in the sense of behavioral addiction and relied on existing behavioral addiction models. They mostly employed the 6-component model and the according measurement [21]. Those components can be criticized, as salience and media use for mood modification need not be problematic. One paper used the behavioral addiction scale and alternated between calling the construct problematic or excessive use [20], writing that it “could be considered a behavioral addiction,” but one should not overpathologize. This back and forth within one study represents the ambiguity in a divided body of literature. Other definitions and scales entail criteria such as excessive use (eg, more use than intended) and difficulty controlling use, but also use to avoid or escape negative emotions and use that creates distress and functional impairment [56].

Third, scholars problematized specific online activities or experiences, for example, paying for Tinder [20], deception [47], abundant availability of partnering options [15,43], chatting with many at the same time, and meeting many new partners (especially after little online communication). In stark contrast, others problematized having few matches and chat conversations [53]. This line of research is in dire need of validated and reliable scales. On the one hand, we need methodologies (eg, experimental) that reveal how many interactions are too little, fine, and too much (and for whom). On the other hand, this shows that the following theoretical questions are not answered: Which activities and which number of options and matches are helpful for relationship seeking, and which number distracts from one’s goal? Which activities are effective for enhancing self-esteem? How many options and interactions [15] are advantageous for well-being? This is very similar to the unresolved question around the ratio of time spent online to offline (another problematized use). Another study examined excessive swiping whereby they (1) defined excessive as compulsive and (2) remained unclear if excessiveness or the activity of swiping was the problem [16].

Fourth, compulsive use of dating apps was problematized. Its dominant characteristic was loss of control over use, in line with definitions in other fields [29]. However, some employed a scale that included use to forget about problems and ill-being if use is not possible [48,53,55]. Coduto et al [45] employed a scale including guilt due to use*.*

Fifth, some scholars problematized a disbalance between the time spent online compared to offline. This topic showed the greatest heterogeneity: On the one hand, some were concerned with a preference for online interactions [45,55] and too little time for face-to-face interactions [45]. Concerns around too many online activities in relation to offline activities mirror the fear that the internet will displace “real” life, that is, traditional ways of living and relating [76]. On the other hand, too little time in online interactions was also considered a kind of problem use of dating apps [51,60]. One study [51] tested if long online interactions, for example, a month or more, correlated with desired outcomes and thereby acknowledged the advantages of longer online interactions. Similarly, having few contacts was an item of self-conscious social comparisons with other users (eg, “I think that most Tinder users have more matches than me”) in the study by Her and Timmermans [53] suggesting that many contacts are preferable. This ambiguity between concerns for too little offline versus too little online time may be explained by the idea that we are concerned about too little offline time with existing ties, but also about too little online time when getting to know strangers.

Sixth, mere use (lifetime or current) or frequency of use was examined by few of the reviewed studies. While there are tons of studies on usage frequency or comparing (lifetime or current) users to nonusers regarding psychological correlates, our search term was supposed to yield only studies mentioning compulsion, addiction, or the like. However, since some studies contained those terms in their outcomes, we had included 3 reports that problematized mere use or mere frequency.

Concerning measurement, the validity of most measures is questionable. In the most frequently used measurement, the criteria conflict and withdrawal [31] entail life problems and emotional reactions [21] and thereby conflate predictor (media use) and outcome. Similarly, use out of ill-being and use that creates life problems [28,45,56] invalidly includes life problems within the measure of media problem use. Including detrimental consequences within the measure of media (problem) use and then using this variable to predict detrimental effects is tautological [25]. Quantitative researchers should not ask participants if they attribute their problems to media use but measure the 2 variables separately and test their association [77]. For future researchers who want to systematize findings, we suggest they only include studies with scales that validly separate problem use from undesired outcomes.

#### Adverse Correlates of Problematized Online Dating

Regarding undesired correlates of problematized online dating, the strongest focus was laid on individual well-being with researchers measuring mood and emotional issues (eg, depressive symptoms) and anxieties. Although psychopathology is a driving force behind addiction, psychopathology at clinical levels remains underresearched (or is explicitly excluded [57]). Self-attitudes such as self-esteem were examined less frequently.

Scholars also tested associations of problem use with user motives and other media variables. Not only was use for certain motives the most frequently employed problem use, but user motives were also examined as predictors of problem use (eg, compulsive use). Research on user motives clearly dominates the field. Importantly, motives are often worded as coping with an undesired state (eg, to forget about problems) or enhancing a desired state (for self-esteem enhancement). Such motives can be expected to correlate with problem use because life problems (eg, low self-esteem) are included within the measure of media problem use (use for self-esteem enhancement motive).

Other media variables were, for example, social media or pornography addiction, which can similarly be expected to yield correlations with problem use due to common method bias (especially if measured with nonvalidated items).

Undesired behaviors such as engaging in a high number of hookups (which some authors conceptualized as an undesired behavior) and neglecting responsibilities were often treated as outcomes of media use. That is, researchers conceptualized dating apps as a contextual factor facilitating undesired behaviors [49,60]. There is a fear that unlimited access to online partnering options may distract from offline duties and facilitate a casual hookup culture. Others claim that internet dating has an “enduring effect” on relationships because couples meeting online stay together for a longer time than those meeting offline [78]. However, an observed difference in relationship length can be attributed to many factors apart from technology: Individuals choosing (paid) online dating services are usually highly motivated to start a relationship and remain partnered [71]. So, the claims that online dating has a causal effect on relationships remain unsubstantiated.

For personality, it is common to use validated scales and to assume the effects of personality on media selection and behaviors, in the sense that (eg, impulsive) individuals choose media in line with their preference (apps affording quick decisions) and use them accordingly (eg, longer than intended). Researchers would not hypothesize that problematic technology use makes people more impulsive.

For partner choice, however, some assume causal effects of dating app problem use. There have been claims that dating app use has revolutionized the way we relate to each other and that, due to an abundance of alternative options, singles may not want to commit and may develop a rejection mindset [79]. While supposedly making connections easier, technology may make it, in fact, more difficult to connect because users may get distracted by too many dating options or by in-app rewards [80]. Yet, this problem has not been quite represented in academic literature.

Sexuality and variables related to interpersonal perceptions and interactions were studied least frequently in the included reports. We lack empirical evidence on the question of whether problem users of dating apps (ie, binge swipers) are also in fact binge daters with plenty of noncommittal hookups. Future research should test if dating app use stimulates (or displaces) in-person dates. Few researchers tested correlations with compulsive sexual behavior, quality of sex life, or intention to commit. This does not mirror the popular fear that online dating (addiction) creates interpersonal problems. If examined, sexual and interpersonal variables tend to be studied as outcomes of online problem behavior [47].

#### Study Designs

An accumulating body of correlational evidence links dating app (problem) use to psychological variables such as self-esteem [13]. Yet, to this date, it remains unclear how they are linked because methodologically, dating app research is not as rigorous as research on other types of media problem use. To address the question of whether dating app (problem) use influences well-being or the other way around [20], we need studies with solid theoretical foundations and strong designs. Moreover, we suggest experimental and longitudinal designs to test causal and over-time effects (between and within subjects). These can tell if problem use of media causally leads to life problems.

### Limitations

It could be that important contributions in this rather young field are about to be published, so the findings of this literature review should be updated regularly. Also, note that samples are not comparable because they had diverse exclusion criteria. For example, psychopathology or substance use were outcomes in one study [57], while exclusion criteria in another study [46].

### Conclusions

The conceptual ambiguity on how problem use is defined restricts the generalizability of findings within the field. The debate around the harmful effects of media (problem) use is ongoing and will probably never be concluded for good because there are too many factors to differentiate (eg, which media, which activities, for whom). Researchers often employ differentiated approaches. However, when reviewing the case of dating apps, research seems to regress to an earlier theoretical stage. We noticed, for example, that studies compare users to nonusers, or that media effects are considered like a hypodermic needle, an outdated theory of media effects theorizing that media stimuli pierce passive audiences like a needle [81]. It seems that with new technologies, the general debate around harmful media effects reignites, and research falls back to earlier stages. Such theoretical and methodological regression is probably especially tempting for emotion-laden topics. Dating and sexuality are highly emotional topics, and technological progress also evokes fear. There is a prevalent fear that new media could become uncontrollable because they are so addictive that (young) users will be unable to regulate their media use [76].

In line with that, we observed the greatest heterogeneity in the question of how online and offline interactions should relate to one another. On the one hand, the advent of modern technologies has often been accompanied by the fear (or even moral panic) that new digital activities will displace traditional offline activities. On the other hand, other researchers prescribe extensive online communication preceding an in-person meeting, hoping that this could remedy dangers of meeting strangers. How online and offline activities relate to one another remains a topic of popular and academic debate.

Overall, theory-based definitions of what constitutes problematic media use, measures that validly separate predictor (media use) and outcome (life problems), and strong designs will improve research on mental health, interpersonal relationships, and technology addiction.

## Supplementary material

10.2196/72850 Multimedia Appendix 1 Additional information about the systematic review of the 29 papers on problematic online dating definitions, correlates, and study designs.

10.2196/72850 Checklist 1 PRISMA (Preferred Reporting Items for Systematic Reviews and Meta-Analyses) checklist.

## Abbreviations

CIUS: Compulsive Internet Use Scale
*DSM-IV*: *Diagnostic and Statistical Manual of Mental Disorders, Fourth Edition*
FB: Facebook
PODAUS: Problematic Online Dating Apps Use Scale
PROSPERO: International Prospective Register of Systematic Reviews
PTUS: Problematic Tinder Use Scale
