# Supplementary material for: Problematic Online Dating: Systematic Review of Definitions, Correlates, and Study Designs
Source: J Med Internet Res. 2025 Jul 3;27:e72850. doi: 10.2196/72850 (PMC12244275; doi:10.2196/72850)
Supplement: Multimedia Appendix 1 [file jmir-v27-e72850-s001.docx]

# Multimedia Appendix

## Methods

We deviated from the registered protocol in two ways: (1) After searching 16 databases, we did not additionally consult Google Scholar; (2) we considered each study equally important and did not assess risk of bias. We collected data on study population (country, age, gender, sexual orientation, user status), definition and measurement of problematic dating behaviors, and adverse correlates. Authors MFT and SD independently collected data from identified records. All records were eligible for each synthesis without preparation. For synthesis, MFT counted and grouped the types of problematic dating behaviors and adverse correlates.

Table S1 outlines each record’s country and study population including sample size, age, gender, sexual orientation, user status, and relationship status. Eight studies use samples from the United States, seven from China or Taiwan, five from European countries (France, Germany, Italy, and Hungary), four from Australia, two from Israel, one from French-speaking Canada, and one sample was recruited online and does not specify country.

**Table S1.** Overview of the country and study population of the 29 papers on problematic online dating (published 2009-2024).

| **Number** | **Reference** | **Country** | **Study population (age, gender, sexual orientation, user status)** |
| --- | --- | --- | --- |
|  | Blake et al [42] | Australia | *N* = 687 (aged 17–51, *M* = 20.28 years, *SD* = 4.63; 23% male; 87% heterosexual; 45% had ever used a dating app; 95% never married) |
|  | Chiou and Yang [43] | Taiwan | *N* = 120 undergraduates (aged 15-23, *M* = 17.49, *SD* = 2.69; 48% males; orientation not reported, 100% had experiences of online romantic relationships) |
|  | Clemens et al [44] | USA | *N* = 678 (aged 18-51 but 97% 18-30; *M* and *SD* not reported, 51% male; sexual orientation not reported although focal variable; 584 undergraduates and 94 recruited from online networking sites; 100% users) |
|  | Coduto et al [45] | USA | *N* = 269 (undergraduates, age range not reported, *M* = 20.85, *SD* = 2.45; 38% male; 91% heterosexual; 100% had lifetime experience with online dating) |
|  | Ding et al [46] | China | *N* = 4057 (age not reported; 33% male; orientation not reported; 100% social media users; online dating user status not reported) First-year students without history of neurological or psychiatric diseases, no use of psychoactive and narcotic drugs and active substances within one week. |
|  | Drouin et al [47] | USA and MTurk | *N* = 272 (aged 18-69, *M* = 32.22, *SD* = 8.32; 52% male; sexual orientation not reported; 55% have ever used online dating) |
|  | Gao et al [48] | China | *N* = 451 (aged 18-35, *M* = 25.17 years, *SD* = 4.25; 49% male; sexual orientation not reported; user status not reported) |
|  | Goedel and Duncan [49] | USA | *N* = 174 (aged 18-58, *M* = 30.80; *SD* = 9.5; 100% male; 100% men who have sex with men; 100% Grindr users); the sample was predominantly non-White and most reported a low income |
|  | Gori et al [50] | Italy | *N* = 384 (aged range, *M* = 25.90, *SD* = 5.21; 34% male; 100% daily dating app users with most using them for up to one hour a day; 73% single) |
|  | Hahn et al [51] | USA | *N*_Study 1_ = 64 (aged 18-24, *M* = 22.65, *SD* = 1.38; 100% male; men who have sex with men of which 15.6% identified as heterosexual; 100% users; 33% single)  *N*_Study 2_ = 217 (aged 18–21, *M* = 20.23, SD = 0.85; 48% male; 69% heterosexual; 59% users; 26% single) |
|  | Harren et al [52] | France | *N* = 825 (aged 18-54, *M* = 25, *SD* = 9.7; 20% male; 82% users; 70% heterosexual 39% single) |
|  | Her and Timmermans [53] | USA | *N* = 296 (18–29, *M* = 26.30; *SD* = 2.90; 61% males; 90% heterosexual; 70% single; 100% current Tinder users) |
|  | Hu [54] | China | *N* = 349 (aged 18-58, *M* = 27.05, *SD* = 4.49; 50% male, 91% heterosexual; 100% used dating apps in past month; 100% single) |
|  | Hu and Rui [55] | China | *N* = 361 (aged 18–60, with 90% aged 18–35, *M* = 29.19, *SD* = 6.02; 44% male; 90% heterosexual; 100% used within past month; 47% not in a monogamous relationship) |
|  | Jayawardena et al [56] | Australia and USA | *N* = 118 (*M* = 33.62, *SD* = 12.67; 100% male; men who have sex with men; 100% had used Grindr within the past 30 days) |
|  | Jennings et al [57] | USA | *N* = 2052 college students (aged 18–57, *M* = 19.92, *SD* = 3.90; 30% male; sexual orientation not reported; 19% users; 52% single, 4% in an exclusive relationship) |
|  | March et al [58] | Australia | *N* = 357 (aged 18–60, *M* = 22.50, *SD* = 6.55; 29% male; 81% heterosexual; 100% had ever used an app) |
|  | Marciano et al [59] | Israel | *N* = 2165 (aged 13-80, *M* = 30.16, *SD* = 10.56, 0% heterosexual, i.e., 49.3% gay men, 21.8% lesbian women; user status not reported) |
|  | Mignault et al [60] | Mostly French Canadians (87.1%) | *N* = 342 (aged 16-29, *M* and *SD* not reported*;* 45% male; 81.3% heterosexual; users and non-users; 68.1% single) |
|  | Orosz et al [61] | Hungary | *N*_Study 2_ = 346 (aged 18-51, *M* = 22.02; *SD* = 3.41; 52% male; orientation not reported; 81% single)  *N*_Study 3_ = 298 (aged 19-65, *M* = 25.09; *SD* = 5.82; 41% male; orientation not reported; 100% had used Tinder in last year; 57% single) |
|  | Portingale et al [62] | Australia | *N* = 296 (aged 18-48, *M* and *SD* not reported; 0% male, 78% heterosexual;  32% were lifetime users, 68% single) |
|  | Rochat et al [63] | Country not collected, recruited through social networking sites | *N* = 1159 (aged 18-74 years, *M* = 30.02, *SD* = 9.19; 53% male; 100% heterosexual; 100% users) |
|  | Rochat et al [20] | Country not collected | This study relies on data from Rochat et al [63]  *N* = 1159 (aged 18-74, *M* = 30.02, *SD* = 9.19; 53% male; 100% heterosexual; 100% users, 66% were in a couple or married) |
|  | Vera Cruz et al [64] | “country of residence were not collected” | Relies on data from Rochat et al [63] but additionally included the non-heterosexuals  *N* = 1387 (aged 18–74, *M* = 29.41, *SD* = 8.98; 49% male; 84 % heterosexual, 100% Tinder users) |
|  | Thomas et al [15] | Germany | *N*_Study 1_ = 667 (aged 18-67, *M* = 41.64, *SD* = 13.26, 50% male; 87% heterosexual; users and non-users; 25 % single)  *N*_Study 2_ = 248 (aged 18-38, *M* = 21.83, *SD* = 3.31; 21 % male; 83% heterosexual; users and non-users; 47% single) |
|  | Thomas et al [16] | Germany | *N* = 464 (aged 16–25, *M* = 21.61, *SD* = 2.48; 47% male; 81 % heterosexual; 100% lifetime users) |
|  | Wu and Chiou [65] | Taiwan | *N* = 128 (aged 18–36, *M* = 25.53, *SD* = 4.82; 54% male; orientation not reported; 100% users of online dating web sites). Within an online-gaming addiction survey. |
|  | Ye et al [66] | China | *N* = 779 (age not reported; 53% male; orientation not reported; probably users and non-users) |
|  | Zerach [67] | Israel | *N* = 347 (age for full sample not reported, between *M* = 24.91, *SD* = 4.94 and *M* = 29.40, *SD* = 6.95 years old; 42% male, 32% heterosexual, bisexuals excluded; 100% active users of Israeli dating websites) |

Considering user status, five papers did not report user status, 18 samples comprised exclusively of users of dating apps, nine samples included both users and non-users (19-82% and three did not report the ratio). Note there is no consensus on what defines a user: Some studies asked for daily [50] or current [53,67] use, others asked for use within the past month [54,56], the past year [61], or lifetime use [16,43,58].

Not all studies reported sexual orientation: Fifteen samples were predominantly (78-100%) heterosexual, nine papers did not report sexual orientation, and five studies focused specifically on LGBTQ+ groups of which three specifically focused on men who have sex with men. Note that there is no consensus on the non-heterosexual orientations, and one study, for example, included homosexual individuals but excluded bisexuals [67], so the numbers are not comparable. Not all papers reported relationship status: Seven^[[1]](#footnote-1)^ samples [15,20,51,52,55,64] had low to moderate proportions of singles (25-47%). Four studies had moderate to high (52-70%) percentages of singles [53,57,60,61]. Only two studies had many singles [54,61]. Note that options are not uniformely defined for relationship status, for example, Jennings et al [57] found that 52% identified as single but then only 4% report to be in an exclusive relationship.

## Results

Table S2 lists each record’s definition of problem use and corresponding measurement. Table S3 lists the adverse outcomes studied, while Table S4 outlines the study designs employed.

**Table S2.** Overview of definitions and measurements of problematic online dating in 29 papers (published 2009-2024).

| **Number** | **Reference** | **Problematized uses of dating apps – definition** | **Problematized uses of dating apps – measurement** |
| --- | --- | --- | --- |
|  | Blake et al [42] | 1) Mere use  2) Motivations for using dating apps | 1) Lifetime use (yes vs. no)  2) Four user motivations: (love, self-worth, ease of communication, and thrill of excitement) measured with items by Sumter et al [68] |
|  | Chiou and Yang [43] | Access to a high number of profiles | Reviewing 40 or 80 profiles (only one profile could be accepted) |
|  | Clemens et al [44] | Gratifications sought on online dating sites | Seven gratifications: finding a relationship; distraction, to create an identity, intercourse, to find a convenient companion, peer pressure/status, and to be social (self-constructed and pre-existing scales) |
|  | Coduto et al [45] | 1) Compulsive dating app use  2) Preference for online social interactions (POSI) via dating apps | 1) Three items adapted from Caplan [27], e.g., “I have made unsuccessful attempts to control my use of dating applications.”  2) Four items adapted from Caplan [27], e.g., “I’m treated better on dating apps than offline.” |
|  | Ding et al [46] | Problematic mobile social media use “refers to an individual’s negative impact on mental health, social adjustment, and daily life as a result of excessive use of the network” | Problematic mobile social media usage questionnaire [69] with 20 items for five dimensions: increased viscosity, physiological damage, misplaced anxiety, cognitive failure, and guilt |
|  | Drouin et al [47] | Internet addiction | 20-item Internet Addiction Test [70], e.g., “How often do you lose sleep due to late-night log-ins?”) |
|  | Gao et al [48] | 1) Motivations (social approval, relationship seeking, sexual experience, pass time entertainment, socializing)  2) Compulsive dating app use: “Compulsive use behavior refers to the inability of individuals to reasonably control their daily behavior, resulting in abnormal consumption patterns.”  3) Subjective online success | 1) Tinder Motives Scale [71]  2) Three items from the Compulsive Use Scale by Dhir et al [72], e.g., “I spend a lot of time thinking about dating apps or planning to use them”  3) Three items by Her and Timmermans [53] |
|  | Goedel and Duncan [49] | Use in a certain mood or for a certain motive | Six contextual factors for app use: when sad, depressed, or lonely; when using alcohol; when using any drugs; when using marijuana specifically; when using other drugs; and to arrange sexual encounters in exchange for money |
|  | Gori et al [50] | Problematic online dating apps use in the sense of a behavioral addiction | Problematic Online Dating Apps Use Scale (PODAUS)^[[2]](#footnote-2)^ |
|  | Hahn et al [51] | Study 1: Meeting after short online contact  Study 2: Mere use (use vs. non-use) | Study 1: Time before meeting (“a few days or less,” “a week,” “a few weeks to a month,” and “a month or more.”)  Study 2: lifetime users (*n*=129) vs. non-users (*n*=88) |
|  | Harren et al [52] | Problematic online dating, similar to problematic social media use, following the six-component addiction model | Problematic Online Dating Apps Use Scale (PODAUS) |
|  | Her and Timmermans [53] | Compulsive: “An abnormality in controlling behavioral consumptions where an individual is unable to rationally manage his/her routined performances” (p. 1305)  Motives; Low online success; making self-conscious social comparisons | Adapted the compulsive use of social media scale [72] by replacing the word “Facebook” with “Tinder,” e.g., “To what extent have you felt an urge to use Tinder more and more?” |
|  | Hu [54] | Compulsive use is “unregulated, excessive uses of Internet-based communication technologies – is one of the most studied concepts of addictive Internet technology use” [27,72]. | Four items from Dhir et al’s [72] Compulsive Use Scale, e.g., “to what extent have you felt an urge to use dating apps more and more?” |
|  | Hu and Rui [55] | Compulsive dating app use | Four items reworded from Dhir et al’s [72] Compulsive Use Scale |
|  | Jayawardena et al [56] | Similarly to a behavioral addiction, encompassing excessive and compulsive use, mood modification, difficulty controlling app use, and continued use of the app despite distress and functional consequences (e.g., Orosz et al [61]). | Adapted Bergen Facebook Addiction Scale^[[3]](#footnote-3)^  User motivations: Use for ease of communication (e.g., “I feel less shy online than offline”) and use for self-esteem enhancement (e.g., “To feel better about myself”) as well as use for escapism (e.g., “So I can forget about school, work, or other things”) and use for companionship (e.g., “So I won’t have to be alone”)  Adapted items from Sumter et al. [68] and from the Facebook Motivation Scale |
|  | Jennings et al [57] | Use for either sexual (*n* = 203) or romantic motivations (*n* = 184) | A single dichotomous item: “If you use any of these [online dating] platforms, have you used them to have sexual encounters with others?” |
|  | March et al [58] | Trolling^[[4]](#footnote-4)^ (commonly including deception, aggression, disruption, and success) | Modified version of Global Assessment of Internet Trolling (GAIT) [73] |
|  | Marciano et al [59] | 1) Compulsive use of dating apps  2) Use for sexual encounters | 1) adapted Compulsive Internet Use Scale (CIUS) [74]  2) Using dating apps for sexual encounters only vs. for other purposes |
|  | Mignault et al [60] | 1) Meeting many new sexual and romantic partners  2) Chatting with many at the same time  3) Seeing people quickly | 1) “Among the new sexual or romantic partners met in the last 6 months, how many were met through a dating app or site, such as Tinder, Badoo, and Happn?”  2) “How many people are you chatting with at the same time?” (on a scale from 1 [one person at a time] to 5 [ten or more])  3) “After how long are you comfortable seeing in person someone met on a dating app or site?” (on a scale from 1 [never] to 6 [after a month or more]). |
|  | Orosz et al [61] | Problematic Tinder use defined on the basis of Griffiths’ [31] six-component model | Problematic Tinder Use Scale (PTUS) |
|  | Portingale et al [62] | 1) Lifetime usage  2) Preferences (e.g., for thinness) | 1) Lifetime usage (yes or no)  2) Among users: six partner preferences^[[5]](#footnote-5)^ |
|  | Rochat et al [63] | Problematic Tinder Use | Problematic Tinder Use Scale (PTUS) |
|  | Rochat et al [20] | 1) Problematic Tinder use (or “excessive” interchangeably)  2) Paying for Tinder  3) Tinder use pattern | 1) Problematic Tinder Use Scale (PTUS)  2) Paying users versus non-paying  3) Mix of motives, number of online and offline contacts, satisfaction, length of use^[[6]](#footnote-6)^ |
|  | Vera Cruz et al [64] | 1) Problematic Tinder use  2) Motives for using Tinder | 1) Problematic Tinder Use Scale (PTUS  2) Motives: enhancement, emotional coping, socialization, finding “true love,” or casual sexual partners |
|  | Thomas et al [15] | 1) Dating app use frequency (incl. non-use)  2) Abundant profile availability (measured in Study 1)  3) Abundant profile availability (manipulated in Study 2) | 1) “How often do you use dating apps?” (incl. “never”)  2) “The number of potential partners is nearly infinite,” “A lot of people come into consideration as a potential romantic partner” in Study 1  3) Three conditions of (11; 31; 91) available profiles in Study 2 |
|  | Thomas et al [16] | Excessive swiping defined as inability to control and compulsivity. (Moderators: modes of decision-making while swiping) | Items based on the excessive smartphone use scale [28].^[[7]](#footnote-7)^ Own items for mode of decision-making while swiping. |
|  | Wu and Chiou [65] | Abundant profile availability | Number of available options (30, 60, 90) |
|  | Ye et al [66] | Social media use frequency for six social media types (messengers, news etc.) | The highest value of daytime and pre-sleep social media use frequency |
|  | Zerach [67] | Cyberbullying victimization and offending | last year experience as a victim and offender of nine different forms of online aggression; four self-generated dating-specific items, e.g., lied to me about age or asked for unprotected sex |

**Table S3.** Overview of adverse correlates of problematic online dating across 29 papers (published 2009-2024).

| **Number** | **Reference** | **Adverse correlates of problem use** |
| --- | --- | --- |
|  | Blake et al [42] | Disordered Eating (Other predictors: Appearance‑based rejection sensitivity; Social rank; Fear of negative evaluation; Emotion dysregulation) |
|  | Chiou and Yang [43] | Excessive searching for the most desirable romantic partner; Difference between stated preference and chosen profile (termed decision quality); Selectivity |
|  | Clemens et al [44] | Big 5; Biological sex; Sexual orientation |
|  | Coduto et al [45] | Adverse outcomes due to dating app use (e.g. missed class or work because of dating apps), negligence in offline social engagements and work-related commitments; Social anxiety |
|  | Ding et al [46] | Alexithymia |
|  | Drouin et al [47] | Deception in online dating; Deception when using sex sites; Perception of others’ deceptive presentation |
|  | Gao et al [48] | Joviality and sadness; Anxiety |
|  | Goedel and Duncan [49] | Risky sexual behaviors (e. g., condomless anal intercourse with one or more partners in the last three months) |
|  | Gori et al [50] | Big 5; Problematic social media use; Problematic cyberpornography use; Love addiction |
|  | Hahn et al [51] | Study 1: Sexual risk behavior  Study 2: Sexual risk behavior; Impulsivity |
|  | Harren et al [52] | Body esteem; Cognitive distraction during sex; Sexual desire; Sexual esteem; Sexual preoccupation; Sexual depression^[[8]](#footnote-8)^ (Three other predictors^[[9]](#footnote-9)^) |
|  | Her and Timmermans [53] | Joviality and sadness; Anxiety |
|  | Hu [54] | Perception that algorithmic recommendation systems restrict user choice; Perceived romantic usefulness of dating apps' matching algorithms; Intention to commit to one person and terminate online dating |
|  | Hu and Rui [55] | Joviality and sadness; Preference for online social interactions  (Moderator: Perceived romantic usefulness of dating apps’ matching algorithms) |
|  | Jayawardena et al [56] | Attachment anxiety; Attachment avoidance; Depression |
|  | Jennings et al [57] | Depression; Anxiety; Insomnia; Suicidal ideation; Internalizing symptoms; Substance use; Sexual risk-taking; Compulsive sexual behavior; Number of lifetime hookups |
|  | March et al [58] | Narcissism; Machiavellianism; Psychopathy; Sadism; Dysfunctional impulsivity |
|  | Marciano et al [59] | Mental distress; Internalized homophobia |
|  | Mignault et al [60] | Risky and unanticipated sexual behaviors^[[10]](#footnote-10)^ and intentions^[[11]](#footnote-11)^ |
|  | Orosz et al [61] | Study 2) Tinder Use Motivation Scale, e.g., self-esteem enhancement; Big5  Study 3) Tinder Use Motivation Scale; Self-esteem; Basic Psychological Need Satisfaction and Need Frustration |
|  | Portingale et al [62] | Appearance-based rejection sensitivity; Body dissatisfaction; Urges to engage in disordered eating; Negative mood |
|  | Rochat et al [63] | Depressive mood; Anxious attachment; Self-control; Sensation-seeking; Having online or offline contacts, number of current matches; Self-esteem; Motives; Dyadic and solitary sexual desire |
|  | Rochat et al [20] | Depressive mood; Happiness; Impulsivity Behavior; Cybersex Motives (enhancement, coping, social) |
|  | Vera Cruz et al [64] | Satisfaction with Tinder app use; Satisfaction with offline dates |
|  | Thomas et al [15] | Study 1: Fear of being single  Study 2: Fear of being single; Self-esteem; Partner choice overload |
|  | Thomas et al [16] | Fear of being single; Upward social comparison; Partner choice overload |
|  | Wu and Chiou [65] | Number of options searched; Average preference difference; Preference difference for the chosen option; Selectivity |
|  | Ye et al [66] | Social media addiction (“excessive and irrational use of social media”); Sleep quality |
|  | Zerach [67] | Pathological narcissism |

**Table S4.** Overview of study designs of 29 studies on problematic online dating (published 2009-2024).

| **Reference** | **Study designs** (*n* = 32) |
| --- | --- |
| Blake et al [42] | Survey |
| Chiou and Yang [43] | Experiment (using real profiles) |
| Clemens et al [44] | Survey |
| Coduto et al [45] | Survey |
| Ding et al [46] | Survey |
| Drouin et al [47] | Survey |
| Gao et al [48] | Survey |
| Goedel and Duncan [49] | Survey |
| Gori et al [50] | Survey |
| Hahn et al [51] | Survey |
| Harren et al [52] | Survey |
| Her and Timmermans [53] | Survey |
| Hu [54] | Survey |
| Hu and Rui [55] | Survey |
| Jayawardena et al [56] | Survey |
| Jennings et al [57] | Survey |
| March et al [58] | Survey |
| Marciano et al [59] | Survey |
| Mignault et al [60] | Survey |
| Orosz et al [61] | Survey |
| Portingale et al [62] | Survey (ecological momentary assessment) |
| Rochat et al [63] | Survey |
| Rochat et al [20] | Survey(same data collection as Rochat et al [63]) |
| Vera Cruz et al [64] | Survey(same data collection as Rochat et al [63]) |
| Thomas et al [15] | Survey and Experiment (using bogus profiles) |
| Thomas et al [16] | Survey |
| Wu and Chiou [65] | Experiment (using real profiles) |
| Ye et al [66] | Survey |
| Zerach [67] | Survey |

1. Note that two of these records report on two samples. [↑](#footnote-ref-1)
2. The PODAUS is an adaptation of the Problematic Tinder Use Scale (PTUS; Orosz et al., 2016). It is a six-item scale used to assess problematic dating apps use (e.g., “During the last year, how often have you thought about online dating sites?”). The six items relate to each of the six different core components of addiction (Griffiths, 2005). [↑](#footnote-ref-2)
3. BFAS; Satici and Uysal, 2015, 18 items [↑](#footnote-ref-3)
4. Provocative, offensive, or menacing online communication, in an attempt to trigger conflict and cause victims distress for the trolls’ own amusement. [↑](#footnote-ref-4)
5. Preference for (1) thinness/slimness, (2) muscle tone, (3) sex appeal, (4) healthy-looking, (5) intelligent-looking, and (6) other [↑](#footnote-ref-5)
6. The number of Tinder-initiated online and offline contacts (from “0 people” to “more than 50 people”); looking for committed romantic partners; looking for sexual partners; the number of current matches indicated on the app; satisfaction with Tinder; time since starting to use Tinder (from “less than 3 months” to “more than 2 years”. [↑](#footnote-ref-6)
7. “I just have to keep swiping - there's no other way”; “I often think about swiping when I'm doing something else”; “I would miss not being able to swipe anymore [↑](#footnote-ref-7)
8. Negative feelings, e.g., “I am disappointed about the quality of my sex life. [↑](#footnote-ref-8)
9. Problematic social media use, problematic online sexual behaviors, sex addiction. [↑](#footnote-ref-9)
10. Lifetime hookups, with someone who had multiple partners or before discussing sexual history or disease status; vaginal or oral sex without condoms or protection against pregnancy and using alcohol or drugs before or during sex; anal sex, fisting, or analingus without condom or adequate protection; having an unexpected and unanticipated sexual experience and leaving a social event with someone just met. [↑](#footnote-ref-10)
11. Going to a social event with the intent to hook up and engage in sexual behavior or sexual intercourse with someone), [↑](#footnote-ref-11)
